# The Acute and Long-Term Effects of Olympic Karate Kata Training on Structural and Functional Changes in the Body Posture of Polish National Team Athletes

**DOI:** 10.3390/sports12020055

**Published:** 2024-02-07

**Authors:** Eliza Gaweł, Anna Zwierzchowska

**Affiliations:** Institute of Sport Sciences, The Jerzy Kukuczka Academy of Physical Education in Katowice, 40-065 Katowice, Poland; a.zwierzchowska@awf.katowice.pl

**Keywords:** spine, range of motion, humeral joint, low back pain, lumbar lordosis, musculoskeletal pain, internal rotation, sports performance, microfet-3, Medi Mouse

## Abstract

The aim of this study was to assess the acute and long-term effects of karate kata training on body posture (range of motion (ROM)) and musculoskeletal pain in elite karate athletes. Twelve kata athletes from the Polish national team participated in the study. A cross-sectional study protocol was used, with direct participatory observation (NMQ-7/6 questionnaire, spinal curvatures and spinal ROM testing, ROM of joints) and natural experiment (225 min of kata training) methods of assessment. Age and number of weekly kata sessions were found to correlate with ROM of the lumbar spine (R = (−0.6), *p* < 0.05). High increase in the prevalence of lumbar hypolordosis and posterior pelvic tilt was noted after karate training sessions. ROM of the inclination in the sagittal plane differed significantly between the first and second trials, by 10.0 degrees on average. Kata stances and their movement pattern seem to be related to the occurrence of disturbances in the ROM of the internal and external rotations of the hip joints and decreased depth of the lumbar lordosis, pelvic tilt, and their ROM. The locations of the long-term musculoskeletal complaints (NMQ-6) seem to result from compensatory changes that occur in the musculoskeletal structures as a result of elite-level kata training.

## 1. Introduction

Among different forms of physical activity, martial arts are known to be a universal sport that stimulates all three groups of motor abilities in preschool and school children [1], i.e., (i) strength and conditioning abilities (strength, endurance), (ii) complex abilities (speed, agility), and (iii) coordination abilities [2]. It also impacts positively on socio-psychological responses and mental health [1]. Even though many combat sports can be listed under the term ‘martial arts’, karate is considered to be one of the most popular combat activities worldwide, both in children and adults [3]. Traditional karate training consists of three main components: (a) kihon/kihon-kata—basic karate techniques, (b) kata—prescribed movement sequences of defensive and offensive techniques, and (c) kumite—fighting with an opponent [4]. All of them are performed in four main Olympic karate styles included in the World Karate Federation (WKF): (1) Shotokan, (2) Shito-Ryu, (3) Goju-Ryu, and (4) Wado-Ryu [5].

Currently, WKF karate competitions are organized in two disciplines, kata and kumite, which are differentiated in terms of the athlete’s physical and physiological profile and characteristics of sports performance [6]. Kata athletes are characterized by a greater contribution of anaerobic endurance and a lower contribution of aerobic endurance compared to kumite athletes [6,7]. Moreover, further differences can be found in the specificity of movement patterns and techniques. Kata is a predetermined sequence of karate techniques performed at maximal intensity, identically under situational conditions, with no interruption from the judges until the end of the kata’s routine [8]. Moreover, a typical kata training contains of four main karate-specific parts, i.e., kihon, kihon-kata, kata sequences, and full kata performance. On the other hand, kumite is an intermittent activity that is interrupted by the judges to announce points/penalties, and both frequency and choice of punches and kicks depends on the technical and tactical skills, pressure of the opponent and/or time, and the score [8]. Also, kumite training includes different karate-training specificity that is mainly focused on defense and offense techniques combined in different tactical combinations.

The crucial element that is related to the effectiveness of kata’s performance is the body posture [9], which is defined as the habitual position of the body that enables the athlete to maintain biomechanical balance during static and dynamic movements [10]. Among different body segments and systems, a crucial indicator of the proper body posture is pelvic tilt and the shape of the anteroposterior spinal curvatures, i.e., kyphosis and lordosis, and the symmetry between each other in the sagittal and frontal planes [11]. Furthermore, the interaction between both static and dynamic stabilization structures impacts the biomechanical range of motion (ROM), both in the spinal curvatures and shoulder and hip girdles [12,13]. Therefore, postural disturbances combined with decreased ROM of joints could reduce the generated level of speed and power of offensive and defensive techniques performed by the karateka.

Even though karate is a symmetrical and complex sport [9], the specificity of kata performance includes also asymmetric movement patterns. Therefore, elite-level athletes may show disturbances both in the muscular and skeletal systems as a consequence of adaptive and thereafter compensatory changes induced by multiple repetitions of the same sport-specific movements (external compensation) [14]. Furthermore, when the basic mechanisms responsible for the musculoskeletal function are disturbed, karatekas are also likely to be predisposed to compensatory and thereafter adaptive changes in the biomechanical structures, which are known as internal compensation [14]. The abovementioned disturbances may also induce specifically-located musculoskeletal pain.

The data related to the body posture (spine) of karate athletes are limited [9,15,16,17]. Some of them suggest the beneficial impact of karate training on the anteroposterior spinal curvatures in karate practitioners [9,15,17], while others point to the disadvantageous effect of karate training on the spinal column [16]. Although the cited studies enable us, in some way, to explain the impact of karate training on body posture, to date there are no studies evaluating this issue in elite karate athletes.

Given the above and the gap in the current scientific literature, it seems reasonable to indicate karate kata training as a crucial factor that may be related to the activation of the body’s compensatory mechanisms that have an impact both on the athlete’s general health and athletic performance. Our previous meta-analysis [14] demonstrated a direct relationship between high-level sport-specific training and postural disturbances in athletes of different Olympic sports. Therefore, it seems justified to perform additional research with the participation of athletes of combat sports such as WKF karate, which is frequently recommended as a physical activity that is a golden mean for correction of postural disturbances and improvement of postural control [9,18,19]. Therefore, the aim of this study was (1) to assess the acute (after kata training) and long-term (years of kata training) effects of kata training on body posture (spinal curvatures, pelvic tilt) and ROM, and (2) to evaluate the prevalence and location of musculoskeletal pain in elite karate athletes. It was hypothesized that kata training induces acute changes in the depth of the anteroposterior spinal curvatures, pelvic tilt, and ROM. Simultaneously, it was hypothesized that long-term elite-level karate training leads to structural changes in the anteroposterior spinal curvatures and ROM of the joints. It was also assumed that musculoskeletal complaints result from adaptive and compensatory changes in the body’s biomechanical structures and are located in the distal parts of the joints of the upper and lower limbs.

## 2. Materials and Methods

### 2.1. Participants

Twelve (female = 4; male = 8; mean age = 17.63 ± 3.36; mean body mass = 62.27 ± 9.87; mean body height = 1.69 ± 0.06) elite WKF karate kata athletes from the Polish national team participated in the study. The inclusion criteria were as follows: (1) elite able-bodied male or female WKF kata athletes (ranks 1–2 of the Polish Karate Union Ranking), (2) at least 13 years old, (3) at least 2 years of training experience at an elite level, (4) at least 3 kata training sessions per week, (5) at least 2 strength and conditioning sessions per week, (6) satisfactory self-reported health status, (7) absence of neuromuscular and musculoskeletal disorders. The exclusion criteria were as follows: (1) non-elite level WKF karate athlete, (2) WKF kumite athletes, (3) the occurrence of an injury in the last two weeks, and (4) withdrawal from the study. The participants competed in the following age categories: (i) U14 (*n* = 1), (ii) cadets (*n* = 3), (iii) juniors (*n* = 4), (iv) U21 (*n* = 1), and (v) seniors (*n* = 3). Moreover, all participants competed in Shotokan style. Table 1 provides a detailed description of the study participants.

All measurements were carried out during a 3-day training camp of the kata national team in the Paralympic Preparation Centre in Wisła. Study participants were allowed to withdraw from the experiment at any time and were informed about the benefits and potential risks of the study. All examinations were performed in the presence of national team coaches and informed consent was obtained from all the participants and from legal guardians of the participants who were under 16 years of age. Furthermore, study participants were instructed to maintain their normal dietary and sleeping habits for 24 h before the examination. The research protocol was approved by the Bioethics Committee for Scientific Research at the Jerzy Kukuczka Academy of Physical Education in Katowice, Poland (No. 9/2012), and met the ethical standards of the Declaration of Helsinki 2013.

### 2.2. Procedures

A cross-sectional study protocol was used in this study, with direct participatory observation and natural experiment methods of assessment [20]. Study participants arrived at the Paralympic Preparation Centre in Wisła on Friday in the morning (10–11 a.m.). First, the participants’ personal data, health information, and karate-related information were collected using a survey questionnaire and personal interview. Next, the assessment of the prevalence and locations of musculoskeletal pain from the last 7 days and 6 months was performed. All physical examinations (anthropometric measurements, depth of spinal curvatures, mobility tests, measurements of the range of motion (ROM) of the joints of the upper and lower limbs, experimental session) were conducted on the following day.

### 2.3. Prevalence and Locations of Musculoskeletal Pain

A procedure proposed by Kuorinka et al. [21] was used to assess the prevalence and locations of musculoskeletal pain from the last 7 days and 6 months using a subjective Nordic Musculoskeletal Questionnaire (NMQ-7, NMQ-6), that included nine body parts: the neck, shoulders, upper back, elbows, wrists, lower back, hips/thighs, knees, and ankles/feet. It should be noted that the NMQ has been found to have a high level of validity (80–100%) and reliability (78–100%) [21]. The questionnaire was completed in the presence of the researcher (EG). Moreover, before starting the examinations study participants were instructed to differentiate two types of the questionnaires in order to report both acute (NMQ-7) and chronic (NMQ-6) musculoskeletal complaints.

### 2.4. Anthropometric Measurements

The somatic characteristics of each participant’s body build and composition were assessed based on a standard procedure used in our previous research [22] and included the following qualities and indices of the body build and composition: body height (BH), body mass (BM), hip circumference (HC), waist circumference (WC), body mass index (BMI), body adiposity index (BAI), and waist to hip ratio (WHR). The measurements of the body’s circumferences were performed with an anthropometric tape in the standard measurement positions [23]. Since karate athletes are regularly (at least each year) subjected to a complex sports medicine examination, the values of BM and BH were collected during the interview based on their medical assessment (athlete’s health form).

### 2.5. Range of Motion Testing (microFET-3 Device)

The assessment of ROM was performed with a hand-held muscle testing dynamometer and inclinometer (microFET-3, Hoggan Scientific LLC. Salt Lake City, UT, USA), which has a 0–180-degree measurement range. Before the measurement, the point of application of the research device was marked with a dark marker on bare skin on the bottom of the measured lever arm. Next, the study participant was placed into a specific ROM position (see Table 2), and the microFET-3 was placed on the marked point, with its sensor mounted perpendicularly to the movement axis (frontal/sagittal/transverse plane). Because of the high sensitivity of the inclinometer’s sensor, the device was reset immediately after placing it on the marked point to enable accurate calibration. Thereafter, the researcher made the first click and saved the angle of the starting position (with an accuracy of 1 degree) on the device. Next, the researcher gave the command ‘GO’ and the study participant performed the movement up to her/his maximal ROM. As soon as the maximal ROM was obtained, the study participant stopped the movement, and the researcher made the second click and saved the angle (with an accuracy of 1 degree) on the device. Next, the researcher made the third click and the microFET-3 device automatically calculated the ROM for the joint tested (with an accuracy of 1 degree). Then, the researcher gave the command ‘RELAX’ after which the study participant returned to their habitual position, while the researcher recorded the obtained value in the study protocol. It should be noted that all measurements were performed by the same researcher (EG) to reduce the risk of error.

To maintain the correctness of the measurement and to minimize the risk of errors, each ROM test was performed three times for each joint tested (separately on left and right sides), and their mean value was used for further analyses. Next, the obtained values of the ROM were compared to the International Standard Orthopedic Measurements (ISOM) ROM classification [23]. Specific characteristics of the testing trials and the initial testing position are presented in Table 2.

### 2.6. Sagittal Spinal Curvatures and Spinal Mobility Testing

Depth of the spinal curvatures in the sagittal plane and the sagittal spinal ROM were assessed with a non-invasible method using a hand-held computer-assisted Medi Mouse device (IDIAG M360) and a standard testing protocol that was specifically explained in our previous study [13]. All measurements were performed by the same researcher (EG) to reduce the risk of error. Moreover, the normative range of the angle of thoracic kyphosis and lumbar lordosis was set at 30 ± 5 [11].

### 2.7. Natural Experiment

The natural experiment started at 7 a.m. and ended at 9 p.m. The protocol included the following stages: (1) morning sagittal spinal curvatures examinations (7 a.m.), (2) warm-up training (15 min), (3) karate kata training—1st session, 90 min—Gankaku), (4) karate kata training—2nd session—Sansai), (5) karate kata training—3rd session, 45 min—Gankaku and Sansai, (6) evening sagittal spinal curvatures measurements (9 p.m.). To assess the effects of elite-level karate kata training on the direct adaptations of the anteroposterior spinal curvatures and their ROM, the measurement with the Medi Mouse device (IDIAG M360) was performed twice: (1) in the morning, before the warm-up training (7 a.m.), and (2) in the evening, after three karate kata training sessions, consistent with the training macrocycle developed by national karate coaches (8–9 p.m.). Each of the training sessions included the following constant karate kata training parts: (1) warm-up (15 min)—mobilization, activation, and speed and power exercises, (2) kihon, kihon-kata (20 min), (3) kata sequences x3 (30 min), (4) full kata performance x2 (20 min), and (5) cool-down (5 min). During the experimental training sessions, study participants performed kihon, kihon-kata, and kata only for two routines, i.e., Gankaku and Sansai.

### 2.8. Statistical Analysis

All statistical analyses were performed using the Jamovi (version 2.2) computer software. The following formula was used to determine the adequate sample size: fpc = sqrt((N-n)/(N-1)), where (i) fpc is the finite population correction factor, (ii) N is the population size, and (iii) n is the sample size. Distribution, homogeneity, means, and standard deviations (SD) of the numerical variables, i.e., anthropometric variables, characteristics of participants (age, sport-specific training experience (years), number of karate and strength and conditioning training sessions per week), numerical values of the spinal curvatures (THa, LLa, Incl, SL, and their ROM in all three analyzed positions) and ROM of the hip (maximal flexion AB/extension/internal and external rotation) and humeral joints (maximal flexion/extension, internal/external rotation)were verified (Shapiro–Wilk test, skewness, kurtosis). Due to the normal distribution, the relationships between the analyzed variables were computed with Pearson’s correlation, except for some variables that did not meet the normality of the distribution, and thus Spearman’s rank-order correlation was applied in those calculations (see Table 3 and Table 4). Moreover, Spearman’s rank-order correlation was used to calculate the correlations between the qualitative variables (NMQ-7/6) and characteristics of participants, spinal curvatures, and ROM (hip/humeral joints). A comparison of the means between the first and second measurements of spinal curvatures was verified using a t-test for dependent samples, and the Wilcoxon test. A two-tailed t-test for independent samples (including Welsch’s test and Levene’s test (for homogeneity of variance)) was performed to determine the differences between males and females. Moreover, a Chi^2^ test of independence (including Cramer’s correlation) was performed to determine the statistically significant relationships between the categorical variables (NMQ-7/6, qualitative characteristic of ROM, and body posture). Correlations were evaluated as follows: trivial (0.0–0.09), small (0.10–0.29), moderate (0.30–0.49), large (0.50–0.69), very large (0.70–0.89), nearly perfect (0.90–0.99), and perfect (1.0) [24]. Confidence interval during the statistical analyses was set at 95%.

## 3. Results

**Figure 1:**
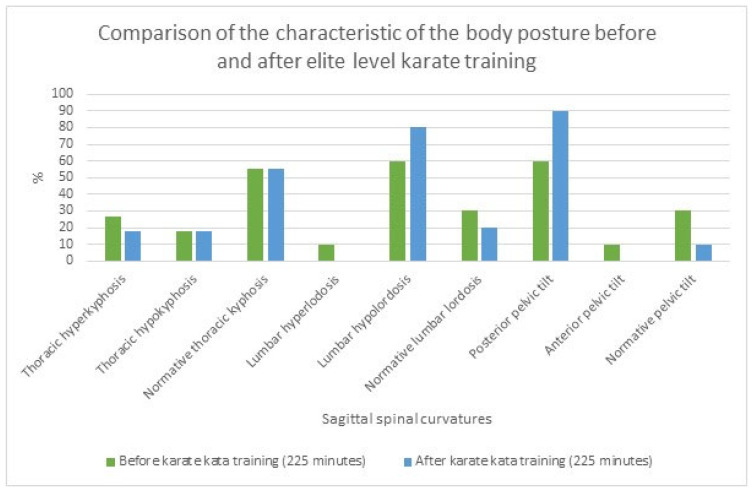
The comparison of the prevalence of the qualitative characteristics of body posture before and after karate kata training (225 min) in elite karate kata athletes based on the Medi Mouse (IDIAG M-360) assessment.

The qualitative (Figure 1) and quantitative (Table 3) characteristics of the anteroposterior spinal curvatures before and after elite-level karate training sessions (225 min) are presented below. In both cases, normal thoracic kyphosis, lumbar hypolordosis, and posterior pelvic tilt were most frequently observed. However, a high increase in the prevalence of lumbar hypolordosis (+20%) and posterior pelvic tilt (+30%) was noted after karate training sessions, which was not observed for thoracic kyphosis as the same prevalence was maintained (see Figure 1).

Pearson’s correlation indicated several statistically significant relationships between the qualitative characteristics of the anteroposterior spinal curvatures, inclination, and characteristics of participants (see Table 3). Interestingly, both internal (age) and external (number of karate kata training sessions per week) variables were found to correlate with ROM of the lumbar spine (R = (−0.6), *p* < 0.05) based on the Spearman’s rank order correlation. Furthermore, the Student’s t-test for dependent samples, including Wilcoxon’s test, indicated that ROM of the inclinationin the sagittal plane differed significantly between the first and second trials, by 10.0 degrees on average. The difference was statistically significant at *p* < 0.02 (Wilcoxon’s test), with an effect size of 0.83 (large).

Table 4 shows the comparison of the ROM of the left and right hip and humeral joints (quantitative assessment) performed with the microFET-3 device. A large decrease in ROM in internal and external rotation of the right and left hip joints and in internal rotation of the left and right humeral joints was observed in karate athletes. Furthermore, Pearson’s correlation between the quantitative variables of the ROM of the humeral and hip joint and ROM of the spinal curvatures (THa, LLa, Incl) and characteristics of participants showed several statistically significant relationships (see Table 4). The analysis revealed some effects of the ROM on the opposite joints. Moreover, similar to the previous results (Table 3), both age and karate training experience were found to be related to the magnitude of ROM in the hip joint (R = (−0.6), *p* < 0.05).

**Figure 2:**
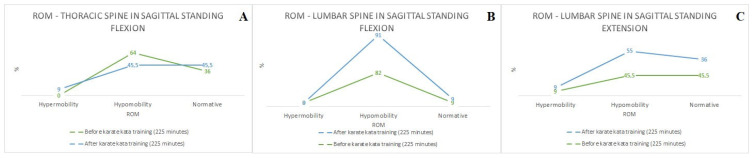
The comparison of the prevalence of ROM between and after karate kata training (225 min) (**A**)—thoracic spine (sagittal standing flexion), (**B**)—lumbar spine (sagittal standing flexion), (**C**)—lumbar spine (sagittal standing extension) (qualitative assessment).

**Figure 3:**
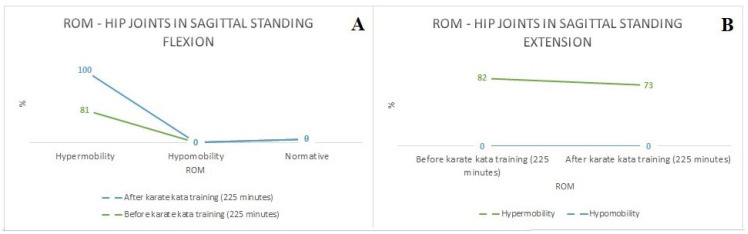
The comparison of the prevalence of ROM between and after karate kata training (225 min) (**A**)—hip joints (sagittal standing flexion), (**B**)—hip joints (sagittal standing extension) (qualitative assessment).

Figure 2A–C presents the comparison of the prevalence of ROM between and after karate kata training (225 min) in (A) thoracic spine (sagittal standing flexion), (B) lumbar spine (sagittal standing flexion), and (C) lumbar spine (sagittal standing extension), while Figure 3A,B shows a similar comparison in (A) hip joints (sagittal standing flexion), and (B) hip joints (sagittal standing extension) based on the qualitative assessment (Medi Mouse (IDIAG M360)). It was found that karate kata training improved the ROM in the thoracic (sagittal standing flexion) and lumbar spine (sagittal standing extension) (9.5%), but it simultaneously decreased ROM in the lumbar spine during sagittal standing flexion (9%) (see Figure 2). For the hip joints, the qualitative analysis indicated that karate kata training increased the hypomobility in the ROM of hip joints (sagittal standing flexion) (19%) and decreased it during performing extension movement in the sagittal plane (9%). Furthermore, regardless of the karate kata training intervention, the majority of karate athletes were characterized by a decreased ROM in the lumbar spine (flexion) and increased ROM in the hip joints (flexion).

**Figure 4:**
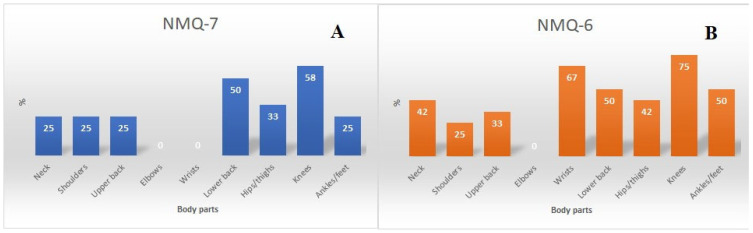
The comparison of the prevalence and locations of musculoskeletal pain based on the NMQ-7 (**A**) and NMQ-6 (**B**) in WKF karate kata athletes (qualitative assessment).

A comparison of the reported musculoskeletal complaints from the last week (NMQ-7) and the last six months (NMQ-6) is presented in Figure 4. Based on the NMQ-6, knees and wrists were followed by lower back and ankles/feet as the most frequently reported locations of pain. Similarly, knees and lower back were reported the most often (NMQ-7); however, surprisingly, wrist pain was not reported by any of the study participants (see Figure 4).

Spearman’s rank order correlation showed an interesting trend, as it indicated several inversely proportional relationships between the prevalence and locations of musculoskeletal pain (assessed by the NMQ-7/6) and ROM in the humeral and hip joints, i.e., neck (NMQ-7) and flexion (A) in the left and right hip joint (R = (−0.6), *p* < 0.05), knees (NMQ-7) and internal rotation in the left (R = (−0.7), *p* < 0.05) and right (R = (−0.7), *p* < 0.01) hip joint, knees (NMQ-7) and extension in the left humeral joint (R = (−0.7), *p* < 0.01), ankles/feet (NMQ-7) and internal rotation in the right humeral joint (R = (−0.6), *p* < 0.05), and neck (NMQ-6) and flexion (A) in the left hip joint (R = (−0.8), *p* < 0.01). Furthermore, upper back pain, assessed by the NMQ-6, was found to show statistically significant relationships both with participants’ age (R = 0.7, *p* < 0.05) and karate kata training experience (R = 0.1, *p* < 0.01).

A Chi^2^ test was performed between the different locations of musculoskeletal pain (NMQ-7/6). The assessment of the body parts based on the NMQ-7 revealed a statistically significant relationship between neck pain and low back pain (LBP), (X^2^ (1, *n* = 12) = 4.0, *p* < 0.05, Cramer’s V = 0.6), while shoulder pain was found to correlate with hip/thigh pain (X^2^ (1, *n* = 12) = 8.0, *p* < 0.05, Cramer’s V = 0.8). Furthermore, several statistically significant correlations were found between body parts assessed by the NMQ-6. Neck pain was found to be correlated to shoulder pain (X^2^ (1, *n* = 12) = 5.6, *p* < 0.05, Cramer’s V = 0.7) and hip/thigh pain (X^2^ (1, *n* = 12) = 5.18, *p* < 0.05, Cramer’s V = 0.7). Shoulder pain also showed statistically significant correlations both with LBP (X^2^ (1, *n* = 12) = 4.0, *p* < 0.05, Cramer’s V = 0.6) and hip/thigh pain (X^2^ (1, *n* = 12) = 5.6, *p* < 0.05, Cramer’s V = 0.7), while knee pain significantly correlated with ankle/feet pain (X^2^ (1, *n* = 12) = 4.0, *p* < 0.05, Cramer’s V = 0.6).

## 4. Discussion

The data on the acute and long-term effects of elite-level WKF karate kata training on athletes’ body posture remain limited. Therefore, this study aimed to assess the abovementioned effects on the depth and ROM of the hip and humeral joints, anteroposterior spinal curvatures, including pelvic tilt, and the prevalence and locations of musculoskeletal pain. As was assumed in our initial hypothesis, this study revealed that elite-level karate kata training (225 min) induces acute changes in the depth of lumbar lordosis and pelvic tilt (decrease), including a decrease in their ROM, while the long-term effects of elite-level karate kata training were mostly a structural lumbar hypolordosis with posterior pelvic tilt and a decreased ROM in internal (humeral and hip joints) and external (hips joints) rotation.

Our findings are consistent with the study conducted by Walicka-Cupryś et al. [16], who indicated that traditional karate training is a factor that contributes to a decrease in the depth of lumbar lordosis and pelvic tilt, leading to the posterior tilt in young and adolescent karate practitioners. Such a decrease in the depth of the lower segments of the spine may be related to karate-specific kata stances that require constant contraction of the flexors of the hip joints [25] to position the pelvis in the posterior tilt, simultaneously affecting the lumbar spine. This effect was particularly observed after performing our experiment as we found a high decrease in the depth of lumbar lordosis and pelvic tilt after karate kata training (225 min) that occurred as adaptive changes in the musculoskeletal structures of the studied karatekas, which points to the external compensatory mechanism.

Furthermore, among different kata stances, zenkutsu-dachi is known to be the most frequent [25]. The movement pattern is focused on maintaining a lowered center of the body’s pressure while bringing the back foot forward, simultaneously causing flexion of the hip joint of the front lower limb and extension in the opposite hip joint [25]. Interestingly, the biomechanical analysis of zenkutsu-dachi (basic kata stance) indicated that kata athletes do not use the biarticular muscles while performing movement patterns, which is related to generating higher moments of muscular force, without converting the body’s potential energy to kinetic energy [26]. This may also be related to the changes in the mechanical properties of the muscles and may lead to structural compensations induced by an external variable, consequently activating internal compensatory mechanisms. Furthermore, these effects may particularly occur during performing repetitive muscle contractions in the kata’s routine. However, from a technical performance perspective, posterior pelvic tilt may have a beneficial effect on maintaining the required position of both karate stances and hips. Nevertheless, such disturbance in the body’s biomechanical system changes the amortization of internal and external biomechanical forces [27], thus causing overload, especially in the lower segments of the spine, simultaneously inducing structural disturbances (flattening of the lumbar spine) and musculoskeletal complaints. Therefore, the results of our study can in some way explain the postural disturbances that have occurred in the lumbar spine and pelvis of elite karate kata athletes, which seems to be a consequence of the specificity of kata stances. This can also be supported by the suggestion of Lisowska et al. [28] that kata stances are related to contractures in the lower limbs, especially in hip abductors.

On the other hand, the vast majority of kata athletes were characterized by a decreased ROM in both the internal and external rotations of the right and left hip joints and by a decreased ROM in the internal rotation of both humeral joints, which also seems to be related to the biomechanics of karate stances, especially zenkutsu-dachi. Similar findings were observed in the study conducted by Polechoński et al. [29], who found a decrease in ROM in the abovementioned joints in young and adolescent kata athletes. However, foam rolling of the lower limbs, especially the quadriceps and tensor fasciae latae muscles, before karate kata training improved the ROM of the analyzed joints. This indicates that a decrease in the ROM in internal/external rotation of the hip/humeral joints is related to the tension of the rotational muscle groups of the aforementioned joints, which seems to be related to the biomechanics of both karate punches and stances and the relationship between them [30]. The complexity of an effective karate punch, i.e., tsuki, is associated with dynamic, internal rotation of the hip joints in the zenkutsu-dachi stance, followed by generation of the maximal velocity of the shoulder with dynamic extension in the elbow joint, after which the hips return to the initial position of external rotation [31]. Such biomechanics and velocity of the movement, combined with flexion of the hip joint and humeral joint of the punching limb and extension of the humeral joint in the opposite limb, could induce both overload and muscular force deficits in the body’s segments and joints, which is consistent with the tendency observed in our statistical analyses, indicating inversely proportional relationships between the prevalence and locations of musculoskeletal complaints and ROM (see Table 3 and Table 4). However, at this point, it should be emphasized that the impact of karate technique on an athlete’s musculoskeletal structure may depend on both the karateka’s strength and kata training experience [32]. Nevertheless, our results indicated that ROM of the hip joint (especially internal rotation) was related to the ROM in the humeral joint, pointing to the complexity of the compensatory processes in both the anatomical trails and the body’s structure.

On the other hand, ROM of the spinal curvatures seems also to be affected by the kata training. Hawrylak et al. [15] found that long-term karate training results in an increase in ROM of the spinal curvatures, excluding the lumbar spine (maximal flexion and extension), in which a decrease in ROM was noted, which is similar to our findings. However, kata athletes in our study were characterized mostly by a decreased ROM, also in the thoracic spine, which may be a result of a high decrease in the depth of lumbar lordosis, especially after kata training. Therefore, it seems that elite-level kata training has a huge impact on the flattening of the anteroposterior spinal curvatures, including their ROM, which could be intensified by the karate training volume [33].

Changes in the magnitude of the depth of lumbar lordosis, pelvic tilt, and ROM of the anteroposterior spinal curvatures and hip and humeral joints are related to different distribution of both internal and external biomechanical forces [24]. As a result, the internal load seems to be located on the distal joints of the karateka’s musculoskeletal system (wrist joint, knee joint, ankle joint). This was indicated in our statistical analyses and revealed as both acute and long-term musculoskeletal complaints, which were simultaneously related to decreased ROM in both humeral and hip joints (see Figure 4—NMQ-6). Therefore, our hypothesis was confirmed. This thesis can be supported by the studies performed by Lisowska et al. [28], who indicated basic kata stances as a variable that induces pain in the joints of the lower limbs and lumbar spine. However, according to the author’s knowledge, to date the available scientific evidence for the abovementioned issue is limited. Therefore, further studies are needed to address the relationship between musculoskeletal complaints and biomechanical karate movement patterns.

### Limitations and Strengths of the Study

The present study has several limitations that need to be addressed. First of all, we investigated a small number of kata athletes (*n* = 12) at various ages and a number of females (*n* = 4) and males (*n* = 8). Secondly, our experiment did not include a post-test of ROM in the humeral and hip joints (performed with a micrFET-3 device), which could improve inference of the acute effects of kata training on the body’s structural parameters. Moreover, study participants specialized only in the Shotokan style. Therefore, our results cannot be transferred to other karate styles such as Shito-Ryu, which are characterized by different kata stances. However, it should be indicated that the study participants were a statistically homogeneous sample of elite-level karate athletes (ranks 1–2 of the Polish Karate Union Ranking). Therefore, it seems to be clear that the group studied was not large. Furthermore, to date, all kata athletes from the Polish Karate Union are Shotokan-style karatekas. Nevertheless, future research should include larger groups of kata athletes from various karate styles and should include post-tests of ROM of the upper and lower body joints.

## 5. Conclusions

1. Elite-level karate (Shotokan style) kata training induces both acute adaptations and long-term structural changes in the position and ROM of the lumbar spine and pelvic tilt;
2. Kata stances and their movement pattern seem to be related to the occurrence of disturbances in the ROM of the internal and external rotations of the hip joints and decreased depth of lumbar lordosis, pelvic tilt, and their ROM;
3. The locations of the long-term musculoskeletal complaints (assessed using the NMQ-6) seem to result from compensatory changes that occur in the musculoskeletal structures as a result of elite-level kata training;
4. Mobility exercises for both internal and external rotation of the humeral and hip joints should be implemented in order to minimalize the risk of musculoskeletal complaints and injury of the abovementioned joints. Simultaneously applying mobility exercises for the lumbar spine could reduce the incidence of low back pain and disfunctions of the lumbar segments of the spine in karate athletes.

## Figures and Tables

**Table 1:** The descriptive statistics and frequency tables of the study participants.

Variables	WKF Karate Kata Athletes (*n* = 12; nF = 4, nM = 8)	WKF Karate Kata Athletes (*n* = 12; nF = 4, nM = 8)	WKF Karate Kata Athletes—Females (*n* = 4)	WKF Karate Kata Athletes—Males (*n* = 8)	WKF Karate Kata Athletes (Males vs. Females)
	Mean ± SD	*p*-Value	Mean ± SD	Mean ± SD	*p*-Value
Age (years)	17.63 ± 3.36	0.26	16.25 ± 2.95	18.25 ± 3.15	0.36
Body Mass (kg)	62.27 ± 9.87	0.41	54.75 ± 4.76	65.75 ± 9.11	0.65
Body Height (m)	1.69 ± 0.06	0.27	1.66 ± 0.04	1.7 ± 0.07	0.24
Hip Circumference (cm)	90.64 ± 6.84	1.0	87.5 ± 5.36	92.13 ± 6.55	0.29
Waist Circumference (cm)	73.73 ± 6.9	0.11	69.25 ± 5.26	75.86 ± 6.09	0.12
BMI	21.65 ± 2.17	0.13	19.95 ± 1.04	22.54 ± 1.93	0.05
BAI (%)	17.73 ± 2.18	0.08	17.29 ± 2.57	18.03 ± 1.76	0.6
WHR	0.81 ± 0.06	0.63	0.79 ± 0.06	0.82 ± 0.05	0.4
Karate training experience (years)	10.82 ± 2.8	0.66	9.25 ± 1.92	11.63 ± 2.64	0.18
Number of karate kata training sessions per week	4.2 ± 1.19	0.04	4.5 ± 1.12	4.5 ± 1.73	1.0
Number of strength and conditioning sessions per week	3.18 ± 1.53	0.005	4.0 ± 2.12	2.75 ± 0.66	0.19

n—total number of participants; nF—number of females; nM—number of males; SD—standard deviation; BMI—body mass index; BAI—body adiposity index; WHR—waist to hip ratio.

**Table 2:** Characterization of the measurements of ROM of the hip and humeral joints, their initial position, and testing movement.

ROM OF THE HIP JOINT		
ROM Trial	Initial Position	Movement
Flexion (A) *	Supine position, both lower limbs straight, upper limbs in the anatomical position	Upright movement of the tested limb up to the maximal ROM, non-tested limb on the floor
Flexion (B) **	Supine position, tested lower limb flexed in the knee joint, non-tested limb straight, upper limbs in the anatomical position	Flexion of the tested limb towards the chest up to the maximal ROM
Extension	Prone position, both lower limbs straight, upper limbs crossed, under the forehead	Upright movement of the tested limb up to the maximal ROM, non-tested limb on the floor
External rotation	Sitting position, both lower limbs over the floor, upper limbs crossed on the chest	Maximal external rotational movement
Internal rotation	Sitting position, both lower limbs over the floor, upper limbs crossed on the chest	Maximal external rotational movement
ROM OF THE HUMERAL JOINT		
Flexion	Habitual standing position, upper limbs straight	Upright movement of the tested limb up to the maximal ROM
Extension	Prone position, both upper and lower limbs straight, forehead touching the mat	Upright movement of the tested limb up to the maximal ROM
External rotation	Supine position, both lower limbs straight, tested upper limb upright, flexed at 90 degrees, non-tested upper limb in the anatomical position	Maximal external rotational movement
Internal rotation	As above	Maximal internal rotational movement

*—maximal flexion of the hip joint was limited by the hamstring muscles, **—maximal flexion was not limited by the hamstring muscles.

**Table 3:** The descriptive statistics of quantitative characteristics of physiological spinal curvatures, inclination, and spinal length in the sagittal plane in three positions (sagittal standing, sagittal standing flexion, sagittal standing extension) in elite karate kata WKF athletes.

Spinal Curvature Measurements: Sagittal Plane	SG (*n* = 11 *) Mean ± SD (°) (First Measurement)	*p*-Value	*p*-Value (Males and Females)	SG (*n* = 12) Mean ± SD (°) (Second Measurement)	*p*-Value	*p*-Value (Males and Females)	Correlation	R–Value (*p* < 0.001 *** p < 0.01 ** p < 0.05 *)
THa—sagittal standing	37.55 ± 14.15	0.98	0.36	36.91 ± 8.75	0.48	0.39	Pearson’s correlation	
THa—sagittal standing flexion	44.27 ± 16.62	0.12	0.64	45.82 ± 17.23	0.33	0.69	ROM—lumbar spine (1) and age	R = (−0.7) *
THa—sagittal standing extension	27.82 ± 18.04	0.15	0.45	25.73 ± 9.8	0.66	0.96	ROM—lumbar spine (1) and WC	R = (−0.7) *
LLa—sagittal standing ****	17.8 ± 16.84	0.09	0.11	9.7 ± 9.34	0.01	0.65	ROM—lumbar spine (1) and WHR	R = (−0.7) *
Lla—sagittal standing flexion	30.91 ± 8.55	0.95	0.2	36.92 ± 12.63	0.67	0.5	ROM—Incl (2) and BAI	R = 0.7 *
Lla—sagittal standing extension	23.45 ± 15.2	0.19	0.86	12.82 ± 10.43	0.05	0.08	Spearman’s rank order correlation	
Incl—sagittal standing **** SL—sagittal standing ****	2.18 ± 1.59 445.4 ± 34.08	0.47 0.64	0.17 0.72	1.64 ± 1.67 429.91 ± 49.75	0.04 0.65	0.3 0.76	LLa (1)—sagittal standing and Incl (2) sagittal standing	R = 0.7 *
Incl—sagittal standing flexion SL—sagittal standing flexion	127.82 ± 8.47 536.73 ± 51.76	0.02 0.48	0.4 0.21	135.18 ± 7.76 528.27 ± 63.77	0.001 0.65	0.35 0.5	ROM—lumbar spine (1) and karate training sessions/week	R = (−0.6) *
Incl—sagittal standing extension SL—sagittal standing extension	51.0 ± 13.03 397.91 ± 35.08	0.87 0.7	0.61 0.6	59.45 ± 11.96 365.91 ± 59.82	0.78 0.55	0.82 0.09		
ROM in the sagittal plane (thoracic spine)	20.27 ± 11.69	0.83	0.2	23.18 ± 18.25	0.11	0.72		
ROM in the sagittal plane (lumbar spine)	53.64 ± 12.51	0.31	0.6	48.09 ± 14.32	0.97	0.09		
ROM in the sagittal plane (inclination)	180.64 ± 18.0	0.17	0.51	194.64 ± 16.76	0.07	0.75		

THa—thoracic kyphosis angle in the sagittal standing; LLa—lumbar lordosis angle in the sagittal standing; Incl—inclination angle in the sagittal standing; SL—spinal length in the sagittal standing; SD—standard deviation; ROM—range of motion;*—*p* < 0.05; **—*p* < 0.01; ***—*p* < 0.001; ****—one study participant was excluded due to error during saving the measurement.

**Table 4:** The comparison of ROM of the left and right hips and humeral joints (quantitative assessment—microFET-3).

		ROM OF THE HIP JOINT						
ROM Trial and ISOM Normal Values (°)	Mean ± SD (LJ) (°)	*p*-Value	*p*-Value (Males and Females)	Mean ± SD (RJ) (°)	*p*-Value	*p*-Value (Males and Females)	Pearson’s Correlation	R-Value (*p* < 0.001 *** p < 0.01 ** p < 0.05 *)
Flexion (A) (125°)	107.33 ± 10.14	0.27	0.01	107.5 ± 14.2	0.99	0.24	RJ flexion (A) (hip) and RJ flexion (humeral)	R = 0.7 *
Flexion (B) (125°)	122.0.3 ± 8.67	0.41	0.34	125.5.38±	0.54	0.83	LJ flexion (B) (hip) and RJ internal rotation (humeral)	R = 0.6 *
Extension (15°)	18.94 ± 4.7	0.001	0.78	18.41 ± 5.58	0.14	0.77	RJ flexion (B) (hip) and age	R = (−0.6) *
External rotation (45°)	33.39 ± 9.53	0.07	0.75	29.5 ± 7.24	0.3	0.46	RJ flexion (B) (hip) and karate training experience	R = (−0.6) *
Internal rotation (45°)	25.33 ± 7.88	0.39	0.76	26.75 ± 9.63	0.06	0.34	RJ internal rotation (hip) and BH	R = (−0.6) *
		ROM OF THE HUMERAL JOINT					RJ internal rotation (hip) and LJ extension (humeral)	R = 0.6 *
Flexion (170°)	174.53 ± 2.66	0.88	0.66	172.67 ± 3.76	0.92	0.89	RJ internal rotation (hip) and RJ extension (humeral)	R = 0.7 *
Extension (50°)	45.53 ± 10.17	0.01	0.51	43.89 ± 7.66	0.94	0.21	ROM THa (1) and LJ internal rotation (hip)	R = 0.6 *
External rotation (90°)	86.55 ± 5.96	0.001	0.08	88.5 ± 3.38	0.001	0.17	ROM LLa (1) and RJ flexion (B) (hip)	R = 0.7 *
Internal rotation (80°)	65.14 ± 12.11	0.45	0.12	61.67 ± 12.84	0.27	0.29	ROM Incl (1) and RJ internal rotation (humeral)	R = (−0.6) *

ROM—range of motion; SD—standard deviation; LJ—left joint, RJ—right joint; THa—thoracic kyphosis angle in the sagittal standing; LLa—lumbar lordosis angle in the sagittal standing; Incl—inclination in the sagittal standing; ROM—range of motion; *—*p* < 0.05; **—*p* < 0.01; ***—*p* < 0.001.

## Data Availability

The data collected and analyzed during the current study are available from the corresponding author on reasonable request.
